# Supplementary material for: Heparin-based hydrogel scaffolding alters the transcriptomic profile and increases the chemoresistance of MDA-MB-231 triple-negative breast cancer cells
Source: Biomater Sci. 2020 Feb 13;8(10):2786–96. doi: 10.1039/c9bm01481k (PMC7497406; doi:10.1039/c9bm01481k)
Supplement: Supplementary file 2 [file BM-008-C9BM01481K-s002.zip › Supplementary File 4/HP-B hydrogel + EGF (s)vControl/Pathways/my_analysis.Gsea.1545201170987/HALLMARK_WNT_BETA_CATENIN_SIGNALING.html]

Details for gene set HALLMARK\_WNT\_BETA\_CATENIN\_SIGNALING[GSEA]

|  || Dataset | expr.class.cls#HYDEGF\_versus\_CONTROL.class.cls#HYDEGF\_versus\_CONTROL\_repos |
| Phenotype | class.cls#HYDEGF\_versus\_CONTROL\_repos |
| Upregulated in class | HYDEGF |
| GeneSet | HALLMARK\_WNT\_BETA\_CATENIN\_SIGNALING |
| Enrichment Score (ES) | 0.27047732 |
| Normalized Enrichment Score (NES) | 0.9211205 |
| Nominal p-value | 0.6013865 |
| FDR q-value | 0.7151741 |
| FWER p-Value | 1.0 |
Table: GSEA Results Summary

  

Fig 1: Enrichment plot: HALLMARK\_WNT\_BETA\_CATENIN\_SIGNALING      
 Profile of the Running ES Score & Positions of GeneSet Members on the Rank Ordered List

  

| PROBE | DESCRIPTION (from dataset) | GENE SYMBOL | GENE\_TITLE | RANK IN GENE LIST | RANK METRIC SCORE | RUNNING ES | CORE ENRICHMENT || 1 | TCF7 | na |  |  | 208 | 2.592 | 0.0728 | Yes |
| 2 | WNT5B | na |  |  | 460 | 2.168 | 0.1297 | Yes |
| 3 | AXIN1 | na |  |  | 912 | 1.742 | 0.1625 | Yes |
| 4 | DVL2 | na |  |  | 1807 | 1.325 | 0.1586 | Yes |
| 5 | LEF1 | na |  |  | 2438 | 1.143 | 0.1627 | Yes |
| 6 | KAT2A | na |  |  | 3035 | 0.996 | 0.1637 | Yes |
| 7 | JAG2 | na |  |  | 3263 | 0.949 | 0.1825 | Yes |
| 8 | PPARD | na |  |  | 3488 | 0.907 | 0.2001 | Yes |
| 9 | MAML1 | na |  |  | 3683 | 0.869 | 0.2181 | Yes |
| 10 | NCOR2 | na |  |  | 3891 | 0.831 | 0.2341 | Yes |
| 11 | NOTCH1 | na |  |  | 4284 | 0.769 | 0.2385 | Yes |
| 12 | MYC | na |  |  | 4314 | 0.764 | 0.2616 | Yes |
| 13 | PSEN2 | na |  |  | 4592 | 0.722 | 0.2705 | Yes |
| 14 | AXIN2 | na |  |  | 5480 | 0.579 | 0.2429 | No |
| 15 | CUL1 | na |  |  | 5551 | 0.570 | 0.2576 | No |
| 16 | FRAT1 | na |  |  | 6195 | 0.477 | 0.2395 | No |
| 17 | SKP2 | na |  |  | 6255 | 0.469 | 0.2515 | No |
| 18 | DKK1 | na |  |  | 7601 | 0.292 | 0.1908 | No |
| 19 | GNAI1 | na |  |  | 7972 | 0.246 | 0.1794 | No |
| 20 | HDAC5 | na |  |  | 8276 | 0.212 | 0.1704 | No |
| 21 | PTCH1 | na |  |  | 9867 | 0.027 | 0.0883 | No |
| 22 | ADAM17 | na |  |  | 10735 | -0.071 | 0.0454 | No |
| 23 | FZD8 | na |  |  | 12693 | -0.312 | -0.0467 | No |
| 24 | CSNK1E | na |  |  | 13033 | -0.346 | -0.0532 | No |
| 25 | NOTCH4 | na |  |  | 14269 | -0.516 | -0.1010 | No |
| 26 | CCND2 | na |  |  | 14645 | -0.580 | -0.1018 | No |
| 27 | HDAC11 | na |  |  | 14772 | -0.599 | -0.0890 | No |
| 28 | FZD1 | na |  |  | 15551 | -0.732 | -0.1060 | No |
| 29 | JAG1 | na |  |  | 16098 | -0.843 | -0.1073 | No |
| 30 | RBPJ | na |  |  | 16927 | -1.063 | -0.1161 | No |
| 31 | TP53 | na |  |  | 17262 | -1.165 | -0.0959 | No |
| 32 | NUMB | na |  |  | 17819 | -1.396 | -0.0799 | No |
| 33 | HDAC2 | na |  |  | 17939 | -1.461 | -0.0389 | No |
| 34 | CTNNB1 | na |  |  | 17952 | -1.465 | 0.0078 | No |
| 35 | NCSTN | na |  |  | 18419 | -1.771 | 0.0406 | No |
Table: GSEA details [plain text format]

  

Fig 2: HALLMARK\_WNT\_BETA\_CATENIN\_SIGNALING      
 Blue-Pink O' Gram in the Space of the Analyzed GeneSet

  

Fig 3: HALLMARK\_WNT\_BETA\_CATENIN\_SIGNALING: Random ES distribution      
 Gene set null distribution of ES for **HALLMARK\_WNT\_BETA\_CATENIN\_SIGNALING**
